# Lung Microbiota and Pulmonary Inflammatory Cytokines Expression Vary in Children With Tracheomalacia and Adenoviral or *Mycoplasma pneumoniae* Pneumonia

**DOI:** 10.3389/fped.2019.00265

**Published:** 2019-06-26

**Authors:** Heping Wang, Qian Zhou, Wenkui Dai, Xin Feng, Zhiwei Lu, Zhenyu Yang, Yanhong Liu, Gan Xie, Yonghong Yang, Kunling Shen, Yinhu Li, Shuai Cheng Li, Ximing Xu, Yongshun Shen, Dongfang Li, Yuejie Zheng

**Affiliations:** ^1^Department of Respiratory Diseases, Shenzhen Children's Hospital, Shenzhen, China; ^2^Department of Microbial Research, WeHealthGene Institute, Shenzhen, China; ^3^Department of Computer Science, City University of Hong Kong, Hong Kong, China; ^4^Department of Respiratory Diseases, Beijing Children's Hospital, Beijing, China; ^5^Institute of Statistics, NanKai University, Tianjin, China; ^6^Department of Pediatrics, Shenzhen Dapeng District Maternity and Child Healthcare Hospital, Shenzhen, China

**Keywords:** adenovirus, bronchoalveolar lavage, cytokine, microbiota, *Mycoplasma pneumonia*

## Abstract

Community-acquired pneumonia (CAP) is a worldwide infectious disease caused by bacteria, viruses, or a combination of these infectious agents. *Mycoplasma pneumoniae* is an atypical pneumonia pathogen that causes high morbidity and mortality in children, and adenovirus can lead to severe pneumonia. However, the etiology of different types of pneumonia is still unclear. In this study, we selected a total of 52 inpatients with *M. pneumoniae* pneumonia (MPP) (*n* = 21), adenovirus pneumonia (AVP) (*n* = 16), or tracheomalacia (*n* = 15) to serve as a disease control. Bronchoalveolar lavage fluid (BALF) samples that had been obtained for clinical use were analyzed. We compared the differences in microbiota and the expression of 10 inflammatory cytokines in samples between MPP, AVP, and tracheomalacia. We found that the bacterial diversity in MPP was lower than that in AVP and tracheomalacia. *Mycoplasma, Streptococcus*, and *Pseudomonas* were predominant in samples of MPP, AVP, and tracheomalacia, respectively. The expression levels of IL-6, IL-8, and IL-10 were significantly higher in inpatients with AVP compared to children hospitalized with tracheomalacia or MPP. The lung microbiota in MPP was remarkably correlated with IL-2, IL-4, IL-5, IL-6, TNF-α, and IL-1α expressions, while this was not found in tracheomalacia and AVP. Microbiota analysis identified a high load of multi-drug resistant *Acinetobacter baumannii* in the lung microbiota of several inpatients, which might be associated with the long hospitalization length and intra-group differences at the individual level. This study will help to understand the microbial etiology of tracheomalacia, AVP, and MPP and to identify effective therapies for these diseases.

## Introduction

Pneumonia presents a high risk of morbidity and mortality in children under 5 years (1, 2). Bacterial pathogens are usually the main contributors to pneumonia incidence and progression, but atypical pneumonia and viral pneumonia are often more severe. In China, *Mycoplasma pneumoniae* pneumonia (MPP) generally results in the highest incidence of atypical pathogen pneumonia (3), whereas adenoviral pneumonia (AVP) represents the highest severity pneumonia in children (4).

A variety of microbial commensals that play an indispensable part in immune education and colonization resistance to respiratory pathogens (5, 6) colonize the respiratory tract. Emerging reports have demonstrated patterns of imbalanced upper respiratory tract microbiota in children hospitalized with pneumonia (7–9). The *Haemophilus-*dominant nasopharyngeal microbiota of patients infected by respiratory syncytial virus are positively correlated with intensive care use and hospital length-of-stay, while *Moraxella-*dominant or *Streptococcus-*dominant microbial profile is associated with chronic or allergic diseases (10). The infrequent *Leptotrichia* rises to the top five genera in patients infected with influenza virus (11, 12). These results imply that imbalanced respiratory microbiota can be pathogen specific. In addition, there is diverse bronchoalveolar lavage fluid (BALF) microbiota between cystic fibrosis, MPP, and *Streptococcus pneunomiae* pneumonia (SPP) patients (13, 14).

Several blood biomarkers have been clinically tested in children with community-acquired pneumonia (CAP), but the etiological diagnosis and estimation of potential outcomes remain unsolved in most cases. Among traditional biomarkers, two host protein assays based on C-reactive protein (CRP) and procalcitonin (PCT) are widely used in pneumonia diagnosis, with PCT being more sensitive to bacterial/non-bacterial cases and for evaluating severity (15). Pavord reported that the blood eosinophil count is related to the patient risk of pneumonia (16). However, none of the known biomarkers can precisely separate bacterial from viral infection and mild from severe disease.

The mucosal immune system of the respiratory tract can secrete various pro- and anti-inflammatory cytokines, including various interleukins (IL), tumor necrosis factor (TNF) family members, and interferon (IFN) to regulate host immune activity, eliminate pathogens, and recover pulmonary homeostasis (17, 18) during respiratory infection. Recent studies have indicated that host immune responses differ according to disease severity and infectious agents during acute respiratory infection (10, 19). The expression of cytokines in pneumonia patients was pathogen specific. According to previous studies, IL-6, IL-8, and IFN-γ represent the lung immune to response to adenovirus infection (17, 20), and IL-1α, IL-2, IL-4, IL-5, IL-6, IL-8, IL-17A, IFN-γ, and TNF-α are the major inflammatory cytokines induced by *M. pneumonia*e infection (17, 21–23). Given the severity and lack of preventive measures in MPP and AVP, it is necessary to explore imbalanced lung microbiota, inflammatory cytokines, and traditional biomarkers to improve clinical intervention.

In this study, we addressed the following two questions: (1) if and how the lung microbiota and immune-related cytokines are altered in children with MPP or AVP, and (2) how the imbalanced lung microecology is associated with pneumonia severity. Fifty-two diseased children were recruited from Shenzhen Children's Hospital, including patients with AVP (*n* = 16), MPP (*n* = 21), and tracheomalacia (*n* = 15), and were analyzed by 16S rDNA and customized cytokine assays.

## Materials and Methods

### Ethics Statement and Informed Consent

This study was carried out in accordance with the recommendations of Declaration of Helsinki, the Ethical Committee of Shenzhen Children's Hospital. The protocol was approved by the Ethical Committee of Shenzhen Children's Hospital under registration number 2016013. Due to the recruited children were <16 years old, their guardians gave written informed consent in accordance with the Declaration of Helsinki.

### Study Subjects and Pathogen Detection

All children with pneumonia were recruited from inpatients in Shenzhen Children's Hospital. Given the disease-specific microbiota (24–26), antibiotic-induced microbiota dysbiosis (27), and the alteration in dominant bacteria by ventilator-associated pneumonia or hospital-acquired pneumonia (28), the exclusion criteria for patients were as follows: (1) other underlying pathologies; (2) antibiotics exposure within 1 month before hospitalization; (3) admitted to a pediatric intensive care unit; or (4) mechanical ventilation during hospitalization. According to the reference standard for the diagnosis of pneumonia (29), the inclusion criteria for children with MPP or AVP were as follows: (1) chest radiograph showing consolidation, atelectasis, or infiltration; (2) diagnosed as *M. pneumoniae* or adenovirus infection via BALF detection (Table 1; Table S1); and (3) no other common respiratory pathogens detected. Considering ethical issues and the invasive sampling method, BALF collection from healthy children is impossible. Instead, children who were diagnosed with tracheomalacia with physical trachea damage and without pathogen infection were selected as control subjects.

**Table 1:** Summary of patients' characters and clinical records.

	**AVP (*n* = 16)**	**MPP (*n* = 21)**	**T (*n* = 15)**	***p*****–value**			
				**Kruskal-Wallis test**	**AVP-vs.-MPP**	**AVP-vs.-T**	**MPP-vs.-T**
**CHARACTERISTICS**							
Gender				0.143	0.571	0.433	0.106
Female	6	11	3				
Male	10	10	12				
Age (years)^#^	1.6 (0.4~4.0)	4.5 (0.8~9.6)	2.5 (0.3~6.7)	0.000	0.004	0.874	0.021
Delivery mode				0.199	0.921	0.171	0.282
Cesarean section	8	9	3				
Vaginally born	8	12	12				
Feed pattern				0.163	0.680	0.045	0.385
Breast feed	14	16	8				
Breast feed + Milk feed	1	1	1				
Milk feed	1	4	6				
Family history of allergy	0	0	1	0.289	1.000	0.483	0.417
History of pneumonia	5	1	3	0.115	0.066	0.685	0.287
Asthma	0	0	0	1.000	1.000	1.000	1.000
**CLINICAL RECORDS**							
Lung consolidation, atelectasis, infiltration	16	21	0	0.000	1.000	0.000	0.000
Hospitalization length (days) ^#^	12.3 (7~21)	9.1 (4~16)	7.7 (2~13)	0.010	0.089	0.040	0.479
Cough	16	19	14	0.620	0.500	0.484	1.000
Fever	16	16	2	0.000	0.680	0.045	0.385
Wheezing	5	2	6	0.024	0.066	1.000	0.029
Fever duration (days) ^#^	16.6 (4~38)	11.8 (0~30)	1 (0~14)	0.000	0.591	0.001	0.008
Mean of peak temperature (°C)	40.1	39.7	39.2	NA	NA	NA	NA
CRP (<0.499 mg/l)	4	5	6	0.549	1.000	0.458	0.465
PCT (<0.5 ng/ml)	5/11^*^	21	3/4	NA	NA	NA	NA
Eosinophil (0.5–5%)	4	12	8	0.549	1.000	0.458	0.465

The normal reference ranges of CRP, PCT and Eosinophil values are <0.499 mg/l, <0.5 ng/ml, and 0.5–5%, according to the laboratory detection. “^#^,” measure with mean (min ~ max); “a/b

* *” means normal/detection number; AVP, Adenoviral Pneumonia; MPP, Mycoplasma pneumoniae Pneumonia; T, Tracheomalacia; CRP, C-reactive protein; PCT, Procalcitonin; NA, Not available*.

BALF samples (three tubes, 1 ml/tube) were collected through fiber optic bronchoscopy (30). One set of BALF samples was immediately sent to the clinical lab for common pathogen detection. *Acinetobacter baumannii, Haemophilus influenzae, Haemophilus parainfluenzae, Moraxella catarrhalis, Staphylococcus aureus, Staphylococcus haemolyticus*, and *S. pneumoniae* were identified by bacterial culture (7). Nucleic acid testing (NAT) was used to diagnose atypical bacterial or virus infection, including that of *M. pneumonia, Chlamydia pneumonia*, adenovirus, respiratory syncytial virus, Epstein-bar virus, cytomegalovirus, or influenza viruses using specific NAT kits (9). The other two sets of BALF samples were stored at −80°C for cytokine assays and DNA sequencing. Sterile gauze, which was used for wiping the tip of the bronchoscope, served as a negative control (31). Saline solutions and DNA extraction kits were also used for contamination assessment.

### Cytokine Immunoassays

We totally select 10 cytokines for detection with the Multiplex Immunoassay Panel (ProcartaPlex, ThermoFisher, Santa Clara, CA, USA), including pro-inflammatory cytokines (IL-1α, IL-2, IL-6, IL-8, IL-17A, IFN-γ, and TNF-α) and anti-inflammatory cytokines (IL-4, IL-5, and IL-10). The supernatant (25 μl) of BALF samples after centrifugation was added to 96-well filter plates and linked to anti-cytokine antibodies following the manufacturer's protocol. The fluorescent signal of each cytokine, indicating its expression level, was identified by a Pixel CCD camera (Luminex Corporation, Austin, TX, USA). The assay results were reported as mean fluorescence intensity (MFI) and were converted to clinically relevant measures (pg/mL) by log transformation according to xPonent® logistic curve fitting (Luminex Corporation, Austin, TX, USA) (32).

### DNA Extraction, Library Construction, and Sequencing

Microbial DNA in BALF was extracted using the Power Soil DNA Isolation Kit (Mo Bio Laboratories, Carlsbad, UK) according to the manufacturer's protocol. The V3–V4 regions of the 16S rDNA gene were selected as the target sequence using 338F-806R primers. DNA amplification was carried out by a PCR kit (AP221-02, TransGen Biotech, Beijing, China) and quantified by Qubit (ThermoFisher, Singapore). The target sequence length was qualified through 2% agarose gel electrophoresis. The qualified DNA was concatenated with the sample-specific dual-index sequences, and the unneeded DNA was filtered by magnetic beads. Then, PCR was used to generate enough DNA for the MiSeq sequencing platform using the V3 sequencing reagent kit (Illumina, San Diego, USA) (33).

### Sequence Processing, Statistical Analysis, and Visualization

The initial sequencing data were processed by the QIIME suite and in-house programs to filter reads as follows: low-quality, error sequences, abnormal read length, redundancies, and chimeric sequences were removed (9, 34). The qualified paired-reads were connected into tags and then clustered into operational taxonomic units (OTUs). The representative OTUs were aligned to the SILVA reference database (v119), and community diversity calculations were conducted following a previous study (14). Principal coordinate analysis (PCA) was used to inspect the divergence of all samples. The confounding effects of children's characteristics on microbial samples were evaluated by PERMANOVA, with the species annotation referring to previous reports (35, 36). Comparisons of patient characteristics and clinical records were performed with the Wilcoxon rank-sum test (continuous variables) and Chi-square test (categorical variables). Comparisons of microbial composition in two groups were conducted using the Wilcoxon rank-sum test, and all multiple testing results were adjusted by the false discovery rate (FDR). Kruskal–Wallis tests were utilized to compare cytokines and dominant genera in BALF microbiota among the MPP, AVP, and tracheomalacia groups. The correlations among clinical characteristics, cytokines expression, and diversity of the bacterial community were computed by linear regression (37). The correlations between microbiota composition and cytokines/CRP/eosinophils were calculated via PERMANOVA. All graphs were produced by “pheatmap” and “ggplot2” of the R software package (v3.2.3).

## Results

### Different Characteristics and Clinical Records in AVP, MPP, and Tracheomalacia Groups

The average age of AVP children (1.6 years) was younger than that of MPP (4.5 years, *p*-value = 0.004), but there was no significant difference with tracheomalacia patients (2.5 years, *p*-value = 0.874) (Table 1). Other characteristics showed no statistical significance between the three groups. AVP children had a higher ratio of abnormal PCT (54.5%) and eosinophils (75.0%) and suffered longer hospitalization length and fever duration as well as higher peak temperature compared to MPP (Table 1; Table S1). When comparing AVP/MPP with tracheomalacia patients, fever duration showed a significant difference (Table 1).

### Sequencing Data Summary; the Disease Group Was the Most Important Factor Associated With Microbiota

A total of 1,379,984 high-quality tags were produced, averaging 26,504 (24,356–28,227), 26,905 (25,392–29,381), and 26,061 (16,894–28,186) for the AVP, MPP, and tracheomalacia groups, respectively. The average OTUs in AVP, MPP, and tracheomalacia groups numbered 616, 469, and 718, respectively. The concentration of DNA extracted from negative controls was lower than 0.01 ng/μl, whereas it was higher than 80 ng/μl in collected BALF samples. In addition, 16S rDNA gene amplification indicated <0.01 nmol/l bacterial DNA in enveloped DNA extraction materials.

Confounder analysis signified that the disease group was the most significant factor to explain variations in microbial samples (*p*-value <0.001), followed by feed pattern (*p*-value = 0.012), gender (*p*-value = 0.018), age (*p*-value = 0.022), and cough (*p*-value = 0.033) (Table S2). The other patient characteristics and clinical records were not strongly related to the microbiota.

### The Lung Microbiota Structure Differed Dramatically Among MPP, AVP, and Tracheomalacia Groups

**Figure 1:**
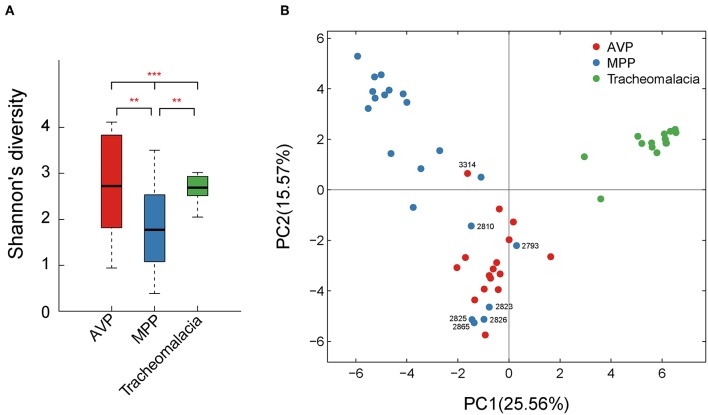
Diversity and principal coordinate analysis (PCA) of lung microbial community differed significantly in adenovirus pneumonia (AVP), *M. pneumoniae pneumonia* (MPP) and tracheomalacia. **(A)** The microbial diversity (Shannon's index) showed significant difference among MPP, AVP, and trancheomalacia. **(B)** PCA exhibited the divergence of microbial samples in three groups. Red, blue, and green represent the microbial samples in AVP, MPP, and tracheomalacia, respectively. ** and *** represent the *p*-value ≤ 0.01 and ≤ 0.001, respectively.

Shannon's index showed that the diversity of BALF microbiota differed significantly (*p*-value = 0.002) in the three groups, and the diversity of the MPP group was lower than those of the AVP group (*p*-value = 0.023) or tracheomalacia group (*p*-value = 0.013), while the lung microbiota diversity in tracheomalacia children was less dynamic than those in the other two groups (Figure 1A). PCA also revealed a divergence among microbial samples in MPP, AVP, and tracheomalacia groups (Figure 1B).

**Figure 2:**
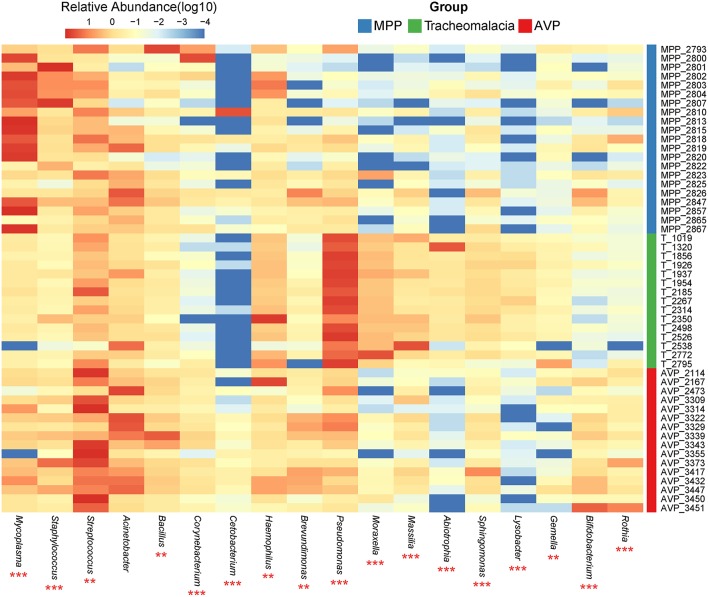
Dominating genera of lung microbiota were discrepant in AVP, MPP, and tracheomalacia. The abundance of dominated genera combined top 10 genera of each group were significant different, except for *Acinetobacter*. The relative abundance was transformed by log10 and proportional to the color from blue to red. Samples from MPP, tracheomalacia and AVP groups were colored blue, green, and red, respectively. ** and *** represent *q*-value of Kruskal-Wallis test ≤0.01 and ≤0.001, respectively.

At the phylum level, Proteobacteria and Tenericutes dominated the lung microbiota in tracheomalacia (74.74 ± 12.20%) and MPP (36.98 ± 32.90%) groups, respectively (Table S3). In contrast, Firmicutes represented the highest abundance in the lung microbiota of AVP children (47.14 ± 32.93%). A comparison of lung-dominating genera among the three groups showed statistically significant differences, except for *Acinetobacter* (*q*-value = 0.053) (Table 2). *Mycoplasma, Streptococcus*, and *Pseudomonas* dominated the BALF microbiota in children with MPP (36.98 ± 32.90%), AVP (36.74 ± 34.59%), and tracheomalacia (43.21 ± 16.84%), respectively (Figure 2; Table 2).

**Table 2:** Comparison of dominant genera (combined top 10 genera) among three disease groups.

	***q*****-value**				**Mean**		
**Dominant genera**	**Kruskal-Wallis test**	**AVP-vs.-MPP**	**AVP-vs.-T**	**MPP-vs.-T**	**AVP**	**MPP**	**T**
*Mycoplasma*	** <0.001**	0.126	**0.003**	**0.001**	1.922	36.979	0.378
*Staphylococcus*	**0.001**	0.570	**0.019**	** <0.001**	2.256	9.391	0.348
*Streptococcus*	**0.003**	0.063	**0.009**	0.793	36.742	4.775	5.315
*Acinetobacter*	0.053	0.241	**0.045**	1.000	12.291	4.483	2.557
*Bacillus*	**0.007**	0.178	**0.003**	0.551	2.965	3.080	0.364
*Corynebacterium*	**0.001**	0.326	** <0.001**	**0.045**	0.470	2.485	0.056
*Cetobacterium*	** <0.001**	0.182	** <0.001**	0.113	0.113	2.011	0.001
*Haemophilus*	**0.009**	0.493	**0.023**	**0.019**	4.243	1.873	6.315
*Brevundimonas*	**0.011**	0.175	**0.007**	0.997	1.586	0.799	0.151
*Pseudomonas*	** <0.001**	0.166	** <0.001**	** <0.001**	2.906	0.786	43.214
*Moraxella*	** <0.001**	0.239	** <0.001**	** <0.001**	0.100	0.247	4.655
*Massilia*	** <0.001**	0.166	**0.017**	** <0.001**	0.474	0.162	3.747
*Abiotrophia*	** <0.001**	0.970	** <0.001**	** <0.001**	0.022	0.025	2.583
*Sphingomonas*	**0.001**	0.296	**0.015**	**0.002**	0.962	0.341	0.902
*Lysobacter*	** <0.001**	0.511	** <0.001**	** <0.001**	0.017	0.009	0.513
*Gemella*	**0.005**	0.959	**0.034**	**0.003**	0.143	0.083	0.507
*Bifidobacterium*	** <0.001**	0.126	** <0.001**	0.291	2.499	0.601	0.048
*Rothia*	**0.001**	0.136	** <0.001**	0.608	1.277	0.364	0.055

*AVP, Adenoviral Pneumonia; MPP, Mycoplasma pneumoniae Pneumonia; T, Tracheomalacia; q < 0.05 are shown in bold*.

### Expressions of Inflammatory Cytokines Differed in MPP, AVP, and Tracheomalacia Children and Were Correlated With Lung Microbiota

**Figure 3:**
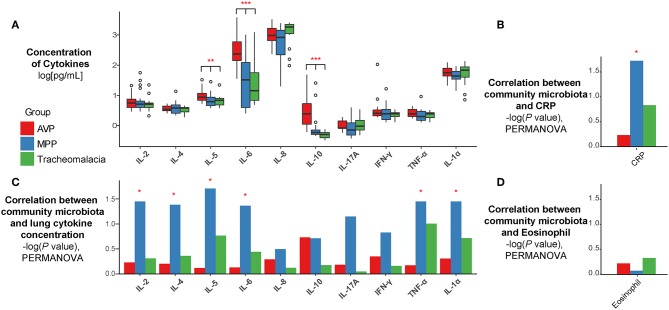
The concentration of cytokines and their correlations with microbiota were disease-specific. **(A)** The expression of cytokines were disease-specific, concentration of cytokines were recalculated by log10 and drawn as boxplot. **(B–D)** The correlation between microbiota and cytokines/CRP/Eosinophil were also disease-specific. Statistical differences were marked as *p*-value on the top of cytokines/CRP. Red, blue, and green represent the patients from AVP, MPP, and tracheomalacia, respectively. *, **, and *** represent q-value of Kruskal-Wallis test ≤0.05, ≤0.01, and ≤0.001, respectively.

The expressions of IL-5, IL-6, and IL-10 in BALF of AVP were significantly higher than those of MPP and tracheomalacia patients (Figure 3A, p-value: 0.016, <0.001, <0.001, respectively). The average expressions of IL-8, IL-17A, and IL-1α were higher, but showed no significant difference in the lungs of children with tracheomalacia compared to those with AVP and MPP. Nevertheless, the expression of IL-8 alone in the tracheomalacia group was significantly different compared to the MPP group (p-value = 0.032).

Based on all samples, the lung microbiota diversity and concentrations of inflammatory cytokines showed no notable correlation (Figure S1). Hence, we then explored the association between the lung microbial community and cytokine expression in AVP, MPP, and tracheomalacia groups. Our results indicated a strong correlation in the MPP group between the lung microbial community and the concentration of six cytokines, IL-2, IL-4, IL-5, IL-6, TNF-α, and IL-1α (p-values = 0.036, 0.042, 0.020, 0.044, 0.036, and 0.036, respectively) (Figure 3B). Additionally, the lung microbiota in MPP was also correlated with serum CRP levels, which were detected within 24 h after hospitalization (Figure 3C). In contrast, we ascertained no significant correlation between lung microbiota and inflammatory cytokines in AVP and tracheomalacia patients (Figure 3B), or between lung microbiota and eosinophil levels (Figure 3D).

### Association of Lung Microbiota and Inflammatory Cytokine Expression With Disease Severity at the Individual Level

**Figure 4:**
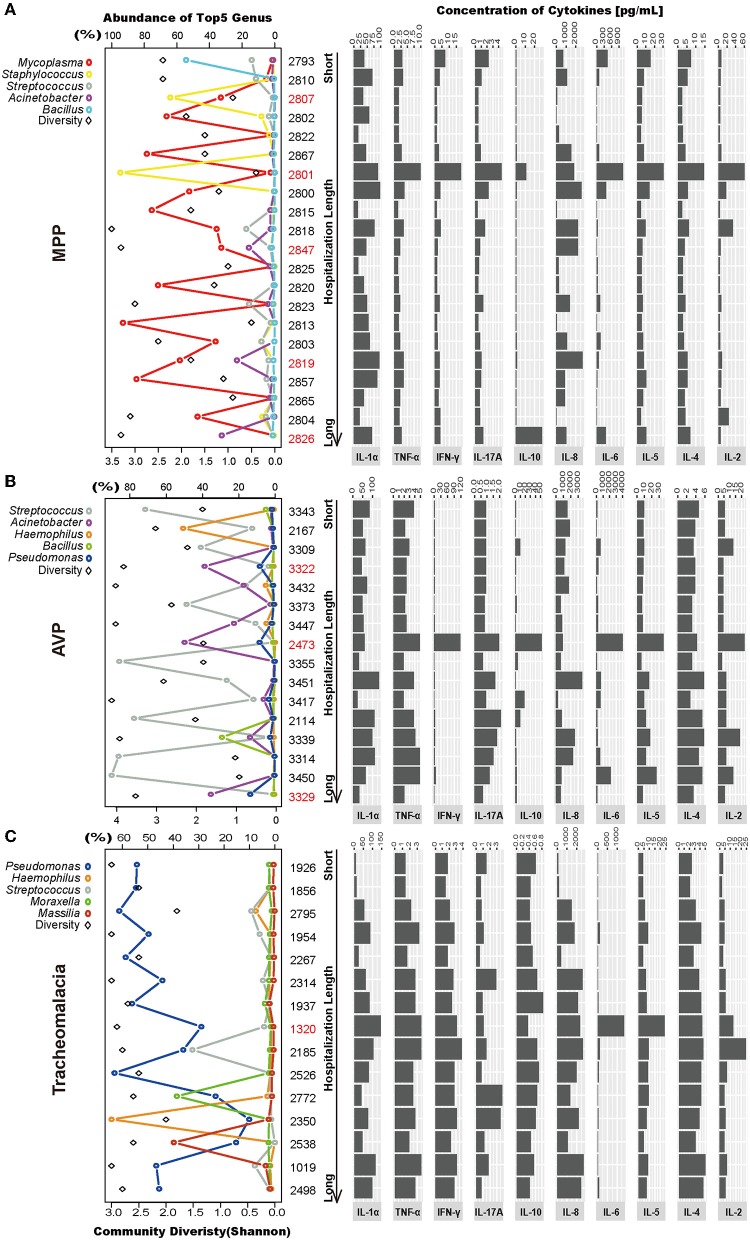
Intragroup microbial structure and cytokines concentration showed individual difference. Top 5 genera and community diversity were used to show community structure in each group (on the left). The concentrations of 10 cytokines were drawn on the right. The patients were sorted by hospitalization length. **(A–C)** Represent the MPP, AVP, and tracheomalacia group, respectively. The top five genera were painted with specific colors and circle, the diversity was shaped rectangle and in black color. Special samples in each group were colored red.

Lung microbiota diversity exhibited no significant correlation with hospitalization length or fever duration (Figure S2). In addition, there was no marked association of CRP, PCT, and eosinophils detected at 24 h after hospitalization with hospitalization length or fever duration (Figure S3). Moreover, at the individual level, we observed wide variability in the lung microbial community and cytokine concentrations in each group (Figure 4).

In the MPP group, the lung microbiota of patients 2,807 and 2,801 was dominated by *Staphylococcus* (64.04 and 94.64%) (Figure 4A; Table S3), which could be assigned as *Staphylococcus warneri* (58.42 and 85.80%, respectively) (Table S4). The bacterial diversity in the lungs was low in both patients 2,807 and 2,801 (Figure 4A). Except for IL-8, IL-10, and IL-1α, the expression of the other seven inflammatory cytokines was the highest in patient 2,801 compared to the other patients (Figure 4A). In the lung microbiota of patients 2,847, 2,819, and 2,826, we identified 15.84, 23.19, and 32.48% abundance of *Acinetobacter* (Figure 4A; Table S3), which was assigned as *A. baumannii* (13.18, 20.76, and 25.20%, respectively) (Table S4). With the increasing load of *A. baumannii* in the lung microbiota, patients 2,847, 2,819, and 2,826 tended to suffer longer hospitalization length (9, 12, and 16 days, respectively) and fever duration (0, 12, and 30 days, respectively) (Table S1). The lung concentration of IL-10 in patient 2,826 (27.89 pg/ml) was dramatically higher than that in the other MPP patients (average of 2.51 pg/ml).

*A. baumannii* was also expected to dominate the lung microbiota in AVP patients 3,322, 2,473, and 3,329 (31.62, 44.55, and 29.76%, respectively) (Figure 4B; Table S4). The expression of five inflammatory cytokines was overwhelmingly higher in patient 2,473 compared to the other AVP patients, whereas patient 3,329 suffered the longest hospitalization length (21 days) (Figure 4B; Table S1). In the tracheomalacia group, patients with *Pseudomonas* dominance (mainly *Pseudomonas veronii*) were inclined to suffer shorter hospitalization lengths (average of 7.1 days) compared to those with other dominant microbial genera in the lung microbiota (9–10 days) (Figure 4C). For patient 1,320, who had the highest lung concentration of IL-6 (1,363.00 pg/ml), the lung microbiota was dominated by *Abiotrophia defectiva* (32.78%) and *P. veronii* (27.26%) (Figure 4C).

## Discussion

A number of recent studies have demonstrated that various microbial commensals colonize the respiratory tract, serving as gatekeepers to respiratory health (38–40). In cases of lower respiratory tract infection, various immune responses can be activated through different pathogens (41), such as pathogen-associated molecular patterns (PAMP) and damage-associated molecular patterns (DAMP) (42, 43). *M. pneumoniae* is a bacterial pathogen that can deplete other bacteria through direct competition or activation of bacterial clearance (17, 18). In contrast, virus infections impair the lung epithelial layer and suppress the immune response, which promotes bacterial outgrowth and frequently leads to secondary bacterial infection (43). These results in lower bacterial diversity in lung microbiota in MPP compared to AVP. Several studies have also reported specific lung microbiota associations, such as Streptococcus being enriched in SPP patients and S. aureus being predominant in patients with methicillin-resistant S. aureus (MRSA) (14, 44). Furthermore, the lung microbiota of children with tracheomalacia is dominated by Pseudomonas, which is consistent with pediatric cystic fibrosis (13). The diversity in lung bacterial compositions can be partly explained by specific mechanisms of pathogen clearance between *M. pneumoniae* and adenovirus.

Though current clinical biomarkers are beneficial for CAP diagnosis, CRP, PCT, and eosinophil counts still have difficulty separating bacterial from viral infections or mild from severe cases. The etiological diagnosis of pediatric CAP and estimations of the outcome remain unsolved problems in most cases. Several cytokines can be induced by *M. pneumoniae*, giving it the potential to predict severity (17, 45). During viral infection, the host immune system is activated through various intercellular signaling cascades (46, 47). IL-6 and IFN-γ are the two major cytokines that inhibit the viral replication and virus-associated inflammation (17, 42, 48). Excessive expression of IL-10 suppresses the clearance of pathogens, which leads to secondary bacterial lung infection (49). In general, clinical biomarkers and cytokines expression can explain the severity of disease in AVP compared to MPP and tracheomalacia. For children with tracheomalacia, combined clinical tests with microbiota and cytokines suggest that lung ecology is shaped by chronic inflammation rather than acute respiratory infection (50, 51). Comparing MPP/AVP with tracheomalacia, the expression of cytokines has the potential to indicate lung microbiota disequilibrium.

Given different genetic backgrounds and living environments (26, 37, 52), homeostasis between microbiota and the immune system in the lungs is individual-specific (37). Current pathogen tests are limited to typically known respiratory pathogens (53) and take a long time to culture, which might mislead or delay pneumonia diagnosis. Microbiota analysis could identify various kinds of bacteria in the meantime, including uncommon respiratory pathogens (54). In this study, several patients were identified with high proportions of A. baumannii, S. warneri, or A. defectiva, which are not detected in conventional respiratory pathogen tests. These highly drug-resistant and pathogenic bacteria (55–57) lead to the overexpression of cytokines and are related to high disease severity (58, 59).

Many studies have sought more robust biomarkers for pneumonia diagnosis. Sonal reports that no single clinical symptom is strongly associated with pneumonia (29). Principi reported that three host protein assays combining CRP, TNF-related apoptosis-inducing ligand (TRAIL), and plasma IFN-γ protein-10 (IP-10) have advantages for pathogen diagnosis and severity assessment over former biomarkers (15). Due to the small sample size and various individual differences, we did not find a strong correlation between clinical information, microbiota, and cytokines in pneumonia.

Several other limitations should be taken into account. Given that no effective medicines exist for MPP/AVP, the patients were provided with empirical treatments, which could disturb the airway ecology slightly (60). Because fiberoptic bronchoscopy is an invasive operation, it is restricted to severe disease cases. Hence, the time points for sample collection were not simultaneous and within a short period after hospitalization. In addition to immune responses, host gene expression should be considered in understanding the pathogen-microbiota-host response axis.

In conclusion, this study provides a significant reference for lung microbiota and immunity in MPP, AVP, and tracheomalacia children. This will fill some knowledge gaps in exploring effective therapies for AVP, MPP, as well as tracheomalacia.

## Data Availability

Sequencing data have been deposited in GenBank under accession number: SRP130820 (Table S5) and SRP138709.

## Ethics Statement

This study was carried out in accordance with the recommendations of Declaration of Helsinki, the Ethical Committee of Shenzhen Children's Hospital. The protocol was approved by the Ethical Committee of Shenzhen Children's Hospital under registration number 2016013. Due to the recruited children were <16 years old, their guardians gave written informed consent in accordance with the Declaration of Helsinki.

## Author Contributions

YZ and DL: conception and design. HW, ZL, GX, and YS: specimens collection and common pathogens detection. XF, DL, and XX: data acquisition and analysis. QZ and WD: result interpretation and manuscript written. YaL, ZY, and YiL: result visualization and revision. YY, KS, and SL: final approval. All authors reviewed this manuscript.

### Conflict of Interest Statement

The authors declare that the research was conducted in the absence of any commercial or financial relationships that could be construed as a potential conflict of interest.

## Supplementary Material

Figure S1 Lung concentrations of cytokines shown no significant association with the diversity of lung microbial community.

Figure S2 No significant correlations between the diversity of lung microbial community with four important clinical records.

Figure S3 No significant correlations between hospitalization length/fever duration and three infection representative blood indictors.

Table S1 The clinical information and cytokines expression of patients with MPP (*n* = 21), AVP (*n* = 16) and Tracheomalacia (*n* = 15).

Table S2 The confounding factors between clinical characters and microbiota by PERAMONVA method.

Table S3 The relative abundance of combined top 10 phylum and genera in MPP, AVP and Tracheomalacia.

Table S4 The relative abundance (%) of species belong to the top 5 genera in MPP, AVP and Tracheomalacia.

Table S5 Released data from our previous study (*n* = 17).
