# Supplementary figures and images for: Lung Microbiota and Pulmonary Inflammatory Cytokines Expression Vary in Children With Tracheomalacia and Adenoviral or Mycoplasma pneumoniae Pneumonia
Source: Front Pediatr. 2019 Jun 26;7:265. doi: 10.3389/fped.2019.00265 (PMC6611399; doi:10.3389/fped.2019.00265)

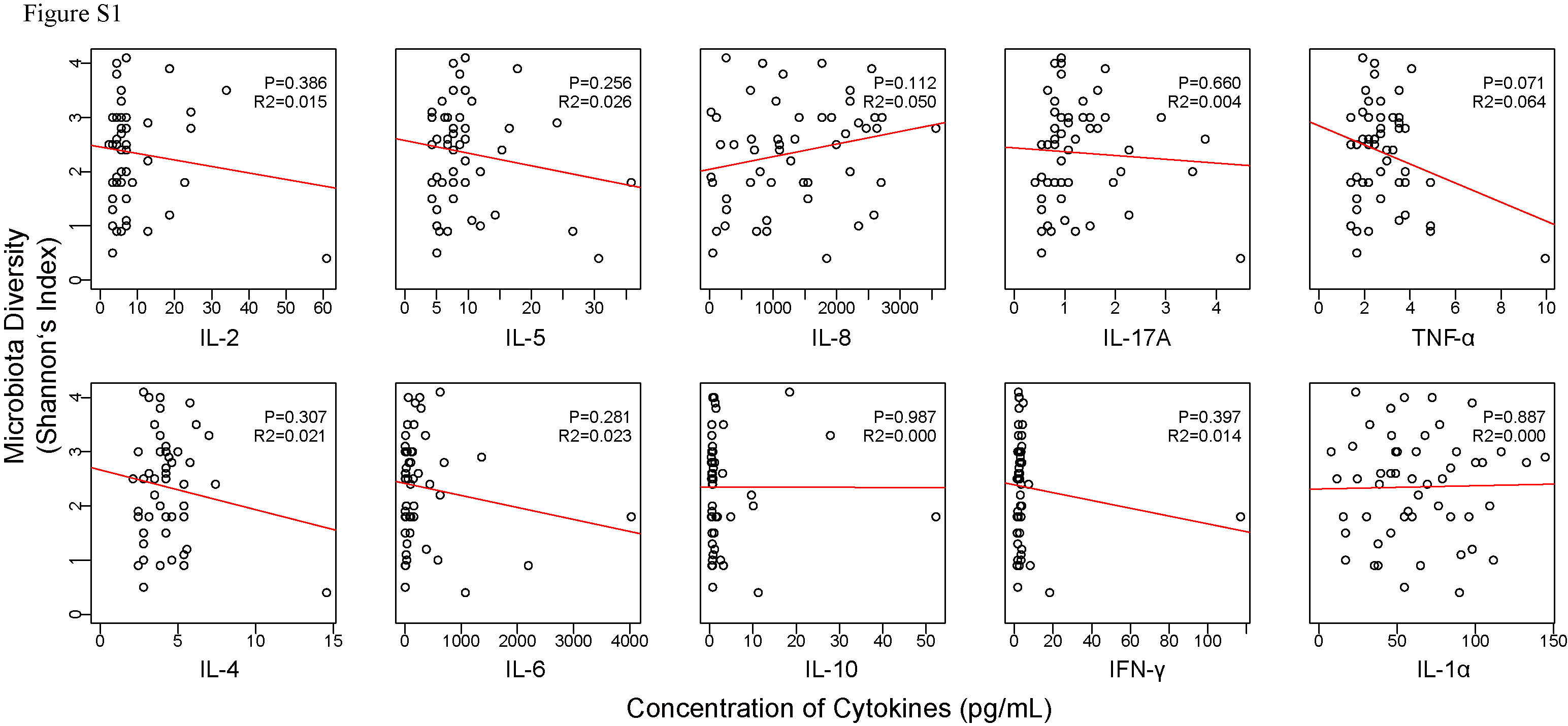

Supplement: Figure S1 — Lung concentrations of cytokines shown no significant association with the diversity of lung microbial community. [file Image_1.TIF]

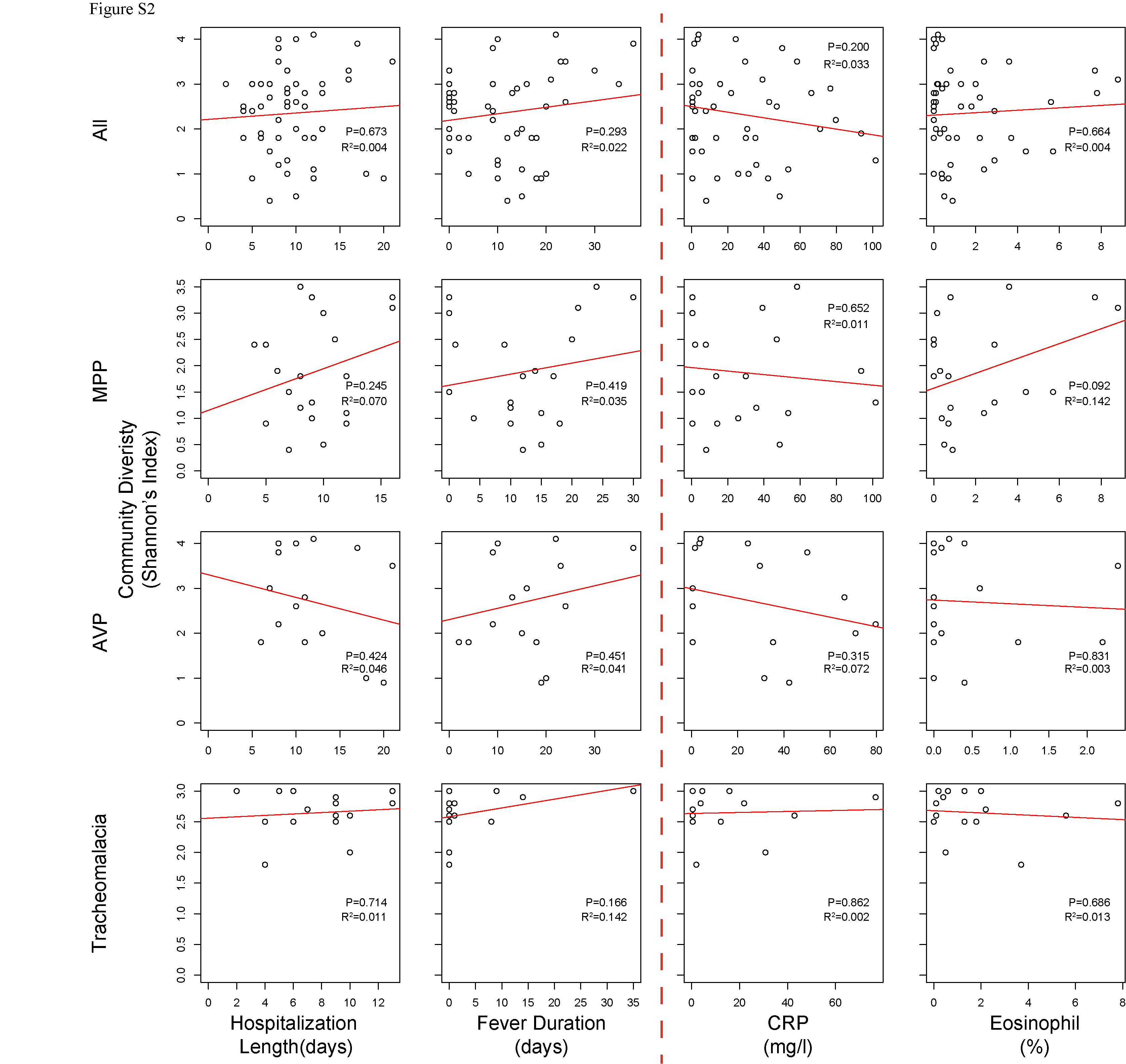

Supplement: Figure S2 — No significant correlations between the diversity of lung microbial community with four important clinical records. [file Image_2.TIF]

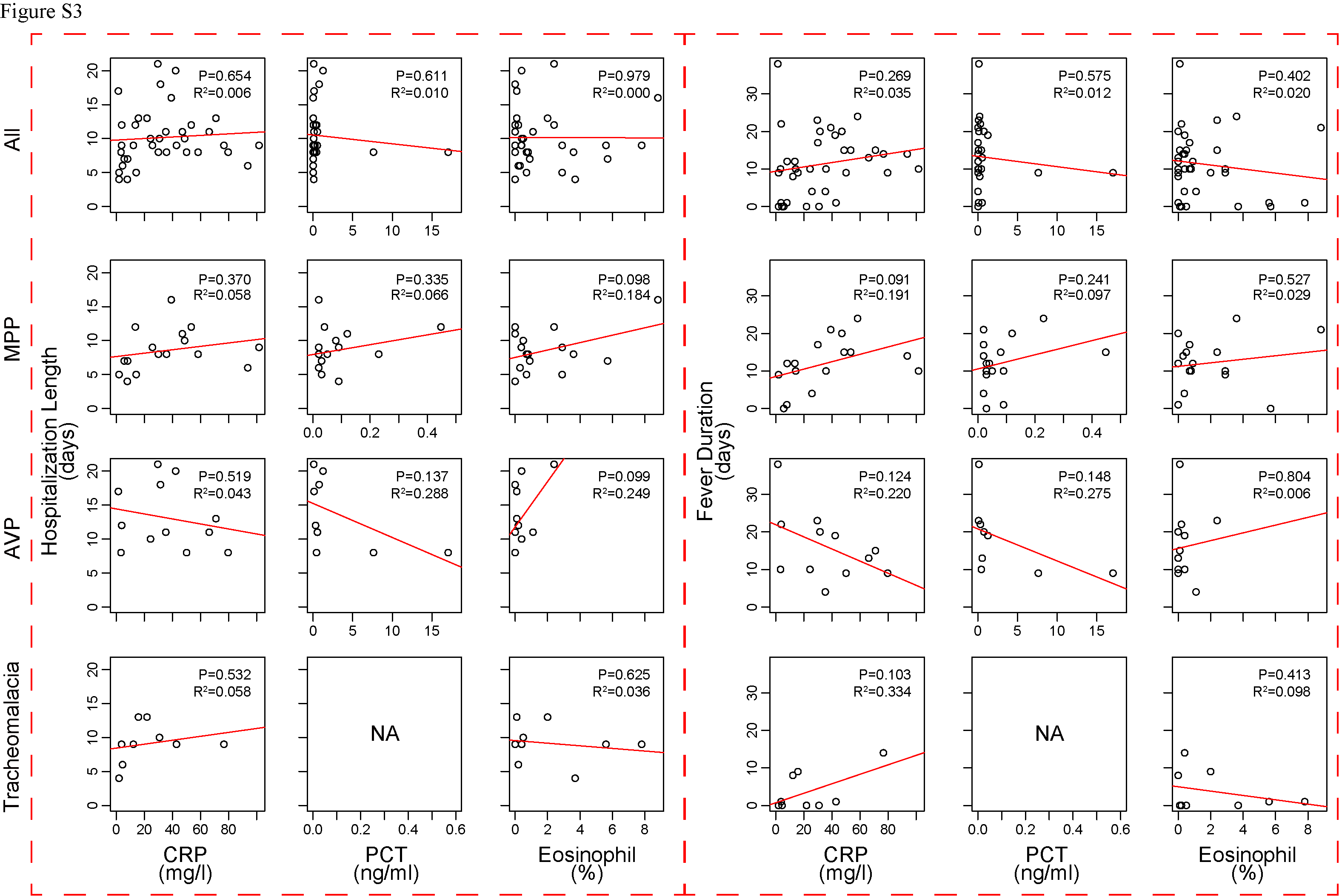

Supplement: Figure S3 — No significant correlations between hospitalization length/fever duration and three infection representative blood indictors. [file Image_3.TIF]
